# Expression of the Endothelin-1 Gene and Its Type a Receptor including Physical Activity among Patients with Acute Myocardial Infarction

**DOI:** 10.3390/ijerph19127289

**Published:** 2022-06-14

**Authors:** Józefa Dąbek, Joanna Piotrkowicz, Joanna Głogowska-Ligus, Małgorzata Domagalska-Szopa, Andrzej Szopa, Lutz Schreiber

**Affiliations:** 1 Department of Cardiology, Faculty of Health Sciences in Katowice, Medical University of Silesia in Katowice, Ziołowa Street 45-47, 40-635 Katowice, Poland; jdabek@sum.edu.pl; 2 Doctoral Studies, Faculty of Health Sciences in Katowice, Medical University of Silesia in Katowice, Medyków Street 12, 40-751 Katowice, Poland; joanna.piotrkowicz@gmail.com; 3 Department of Epidemiology, Faculty of Health Science in Bytom, Medical University of Silesia in Katowice, Piekarska 18, 41-902 Bytom, Poland; 4 Department of Medical Rehabilitation, Faculty of Health Sciences in Katowice, Medical University of Silesia in Katowice, Zapolskiej Street 3, 41-218 Sosnowiec, Poland; mdomagalska@sum.edu.pl; 5 Department of Physiotherapy, Faculty of Health Sciences in Katowice, Medical University of Silesia in Katowice, Medyków Street 12, 40-751 Katowice, Poland; aszopa@sum.edu.pl; 6 Department of Neurosurgery Klinikum Vest, Academic Teaching Hospital, Ruhr University Bochum Germany, 44801 Bochum, Germany; lutz.schreiber@klinikum-vest.de

**Keywords:** physical activity, myocardial infarction, genes

## Abstract

Cardiovascular diseases are the most common causes of death, in both Poland and the world. Their development and progression are largely influenced by the lifestyle with the presence/occurrence of classic, modifiable risk factors. Among them, low physical activity plays a significant role. The aim of the study was to evaluate the expression of the endothelin-1 gene and its type A receptor, taking into account physical activity (International Physical Activity Questionnaire—IPAQ) among patients with acute myocardial infarction. A total of 234 patients with acute myocardial infarction were examined, including 167 patients undergoing early post-hospital cardiac rehabilitation and 67 not participating in it. All of them were assessed with the IPAQ questionnaire and the quantitative real-time polymerase reaction method (QRT-PCR). Physical activity in the group of patients after early post-hospital cardiac rehabilitation increased after rehabilitation. Transcriptional activity of the endothelin-1 (ET-1) gene in both studied group of patients increased significantly, but in a group of patients not participating in early post-hospital cardiac rehabilitation more than in a group of patients participating in it. In our study, the expression of ET-1 was also significantly higher in the group of patients with acute myocardial infarction with ST-segment elevation, without diabetes, with lipid disorders, smoking, with normal body weight. Expression of the ENDRA (Endothelin receptor A) gene increased with age. These results prove the beneficial effect of rehabilitation and may indicate another pathomechanism of pro-atherogenic activity of above-mentioned factors.

## 1. Introduction 

Cardiovascular diseases are the most common causes of death, in both Poland and the world. Their development and progression are largely influenced by the lifestyle with the presence/occurrence of classic, modifiable risk factors. Among them, low physical activity plays a significant role [1,2,3].

Scientific research emphasizes the beneficial effect of exercise on the human body [4,5,6]. Regular physical activity improves different aspects: vascular endothelium function, glucose tolerance, lipid profile, exercise capacity, stroke volume, and cardiac output. Moreover, regular physical activity normalizes blood pressure, heart rate, regulates peripheral vascular resistance and blood flow distribution [7,8].

The vascular endothelium plays an important role in the development of cardiovascular diseases, especially coronary artery disease, heart failure, hypertension, as well as diabetes, and hyperlipidemia. Numerous scientific studies suggest that endothelial dysfunction is the initial stage of atherosclerosis development, preceding the occurrence of morphological vascular changes and clinical disease symptoms [9,10,11].

The risk factors may lead to the endothelial functions’ failure and intensification of the pro-inflammatory, pro-adhesive and pro-thrombotic reactions and consequently to the development of coronary atherosclerosis. The progression of atherosclerotic lesions is associated with the accumulation of cholesterol particles, migration of monocytes and their differentiation into macrophages, foam cells creation, LDL cholesterol particles oxidation, the presence of proinflammatory cytokines, migration of smooth muscle cells to the inner vessel wall and aggregation of platelets. All the above-mentioned processes lead to the formation of atherosclerotic plaques, their growth, rupture, and consequently—A heart attack [12]. Restoring the proper functioning of the vascular endothelium by eliminating modifiable risk factors may inhibit the development of the disease, its progression, and reduce morbidity and mortality [13].

Endothelin-1 (ET-1; endothelin-1) is a 21 amino acid peptide and under physiological conditions, secreted by endothelial cells in small amounts, acting as an auto and/or paracrine mediator. It is produced from preproendothelin and big endothelin with the use of endothelin converting enzyme (ECE) [14]. 

The factors stimulating the secretion of endothelin-1 include: reduction in endothelial shear stress, adrenaline, thrombin, angiotensin II, hypoxia, vasopressin, interleukin-6, insulin, transforming growth factor-beta, homocysteine, calcium ions, and free radicals. On the other hand, the factors that inhibit its release include: Nitric oxide, prostacyclin, atrial natriuretic peptide, heparin, and prostaglandins [15,16].

Under pathophysiological conditions, endothelin-1 shows vasoconstrictor, mitogenic, pro-inflammatory, and pro-thrombotic effects, and its excessive secretion contributes to the development of cardiovascular diseases such as: atherosclerosis, arterial and pulmonary hypertension, heart failure, and diabetes [17]. Endothelin-1 contributes to the development of oxidative stress. Increased expression of ET-1 and its receptors leads to the excessive production of reactive oxygen species, in particular O^2−^, and a reduction in the bioavailability of nitric oxide. As a result of the reduction in NO concentration, the vasomotor functions are impaired with impaired vasodilating functions of the endothelium, which is manifested by excessive vasoconstriction [18].

Endothelin-1 is secreted primarily by endothelial cells, but its expression has also been detected in macrophages and leukocytes. Scientific research indicates that endothelin-1 is a mitogenic factor for smooth muscle cells and macrophages [19,20]. Since all these cell types are involved in the formation of atherosclerotic plaque and in the development of coronary artery disease, it has been suggested that the increased plasma concentration of endothelin-1 and its presence in the macrophage-rich plaque triggers and maintain the inflammatory processes associated with its development. Moreover, it was noticed that among people with chronic kidney disease, increased endothelial ET-1 expression contributes to the progression of atherosclerotic lesions by stimulating lipid biosynthesis and is a prognostic factor for the development of atherosclerosis [21]. 

Endothelin-1 acts through two receptors: type A (ENDRA; endothelin receptor A) and type B (ENDRB; endothelin receptor B).

It was noticed that both the increase in endothelin-1 expression and of its binding with receptors, especially type A, contributes to the progression of atherosclerotic lesions. Endothelin receptor A is located in smooth muscle cells and is mediated under physiological conditions in most reactions related to vasoconstriction, and in pathological conditions, it is responsible for their strong contraction, cell proliferation, and pro-inflammatory effect. The stimulation of the ENDRA as a result of the action of endothelin-1 in smooth muscle cells increases the binding of proteoglycans with LDL particles, contributing to the development of hypercholesterolemia. Published research results suggest that blocking the endothelin receptor A affects endothelial-dependent vascular relaxation as a result of the increased availability of nitric oxide [22]. It also inhibits the progression of atherosclerotic lesions by limiting the formation of macrophages and oxidized LDL particles and the infiltration of monocytes. Moreover, among patients with hypercholesterolemia, it increases blood flow through the vessels [23].

The aim of the study was to evaluate the expression of the endothelin-1 gene (ET-1) and endothelin-1receptor type A (ENDRA), taking into account physical activity (International Physical Activity Questionnaire—IPAQ) among patients with acute myocardial infarction.

## 2. Material and Methods

### 2.1. Participants

A total of 234 patients with acute myocardial infarction were examined, including 167 patients undergoing early post-hospital cardiac rehabilitation and 67 not participating in it. The examined patients were divided into groups:

- A—Which included 167 patients after acute myocardial infarction, who were then referred for early post-hospital cardiac rehabilitation. Group A patients were examined twice: before starting rehabilitation (mean 7–14 years days after discharge from hospital), (A) and after its completion (after about 3 weeks) (A’).
- B—Which included 67 patients after acute myocardial infarction not participating in cardiac rehabilitation. These patients were also examined twice: on average, 7–14 days after being discharged from the hospital (B) and about 3 weeks after the first examination (B’).

All patients gave their informed consent to participate in the study. The study was approved by the Bioethics Committee of the Medical University of Silesia (KNW/0022/KBI/98/15;KNW/0022/KB1/69/18). The study inclusion criteria were: confirmed acute myocardial infarction and informed, voluntary consent of the patient to participate in the study. The exclusion criteria were: acute and chronic inflammation, autoimmune diseases, renal failure, neoplastic disease, and the patient’s serious condition. General characteristic of the studied group of patients after myocardial infarction including sex, age, weight, height and BMI are presented in Table 1.

### 2.2. Methods

All patients included in the studied group were interviewed for risk factors and a transthoracic echocardiogram (UKG), 12-lead resting electrocardiogram (ECG), and some laboratory tests were performed: markers of myocardial necrosis, lipid profile, blood count, serum creatinine, glucose, and electrolytes. 

5 mL of peripheral blood from the antecubital vein of all patients on an empty stomach, was collected twice (that is: 7–14 days after discharge from the hospital and about 3 weeks after the first examination). 

The collected blood was used to examined the expression of the endothelin-1 gene and its A-type receptor by quantitative real-time polymerase chain reaction (QRT-PCR) [24]. It was carried out in the following stages: obtaining material for research, extraction of nucleic acids, qualitative and quantitative evaluation of RNA extracts, and quantitative polymerase chain reaction in real-time. The transcriptional activity of the studied genes was inferred from the number of mRNA copies per 1μg of total RNA.

To assess the physical activity of the studied patients, the 7-day version of the International Physical Activity Questionnaire (IPAQ) was used. IPAQ is a standardized, widely used, and available research tool that determines physical activity in MET units (metabolic equivalent). It consists of 4 basic domains: hard work, moderate work, walking, and time spent sitting during the day. In the first part of the questionnaire, the respondents specified the number of days and time spent during the last week on efforts requiring increased heart rate and breathing, such as aerobics, fast running, and fast cycling. Heavy efforts are assigned with a MET value of 8. To calculate the hard work factor (AF1), the following formula was used:

AF1 [MET] = 8 MET × number of days in the week × amount of time [min.]

Abbreviations explanation: AF1—Hard work factor; MET—Equivalent metabolic; min.—Minutes.

Moderate effort factor (AF2) such as cycling at a normal pace, playing volleyball or brisk walking, assigned a value of 4 MET, was calculated by the formula:

AF2 [MET] = 4 MET × number of days in the week × amount of time [min.]

Abbreviations explanation: AF2—Moderate work factor; MET—Equivalent metabolic; min.—Minutes.

The third part of the questionnaire concerned the time spent walking during the day (AF3). Efforts of nature such as walking or walking to work or shopping, and lasting at least 10 min at a time, were assigned with a value of 3.3 MET. The factor was calculated according to the formula:

AF3 [MET] = 3.3 MET × number of days in the week × amount of time [min.]

Abbreviations explanation: AF3—Light Work Factor; Met—Equivalent metabolic; min.—Minutes.

In the last part of the questionnaire, the respondents determined how much time during the day they spent sitting, e.g., at work, watching TV, taking into account weekdays. 

Based on the above data, patients were classified into a group with a high, sufficient, or insufficient level of physical activity [25].

### 2.3. Intervention

Examined patients (group A) participated in nationwide comprehensive cardiac rehabilitation program (KOS-ZAWAŁ—Comprehensive Patient Care after a Heart Attack). Other patients (group B) did not participate in it due to a serious health condition, numerous comorbidities that prevent the patient from functioning independently and lack of consent to participate in early post-hospital cardiac rehabilitation. 

Hospitalization after myocardial infarction lasted approximately 4–5 days. During above-mentioned hospitalization all patients underwent rehabilitation in a hospital ward consisting of simple breathing and circulatory exercises, as well as standing up and walking. 

On average, 7–14 days after discharge from the hospital, patients participating in early post-hospital cardiac rehabilitation (group A) performed an exercise test on a treadmill, on the basis of which they were qualified for the appropriate model of cardiac rehabilitation:

A model—Patients with good exercise tolerance (≥7 METs); recommended training: continuous endurance training, resistance training and general fitness exercises with an intensity of 60 to 80% of the heart rate reserve for 5 days a week, the exercise duration is 60 to 90 min a day,

B model—Patients with good and moderate exercise tolerance (≥5 METs); recommended training: Continuous or interval endurance training with an intensity of 50–60% of the heart rate reserve, 45–60 min a day, resistance training for 2–3 days a week, and general fitness exercises for 5 days a week,

C model—Patients with low exercise tolerance (3–5 METs); recommended training: interval endurance training and general fitness exercises (5 days a week), elements of resistance training (low resistance, 2–3 days a week), 45 min a day, and its intensity should be between 40 and 50% of the heart rate reserve,

D model—Patients with very low exercise tolerance (<3 MET); recommended training: individual exercises ranging from 30 to 45 min, the intensity of the exercise should be below 20% of the heart rate reserve or cause the heart rate to accelerate by no more than 15–20%.

The characteristics of the studied group of patients after myocardial infarction participating in early post-hospital cardiac rehabilitation (A), including the rehabilitation model, is presented in Table 2.

Mentioned patients participated in training sessions 5 times a week. Each session consisted of two parts: 

1. Training on a bicycle ergometer—Continuous (A model) or interval (B or C model), depending on the patient’s exercise tolerance, and the training duration ranged from 15 to 30 min, depending on the rehabilitation model.

2. Group exercises in the gym, lasting 30 min. This training began with a warm-up, which the purpose was to prepare the movement, circulation and respiratory systems for physical exertion. In the main part, which lasted from 15 to 20 min, the patients performed slow active exercises, with resistance, general fitness and breathing exercises. In order calm down the body after the effort, low-load, stretching, relaxation and breathing exercises were performed. 

### 2.4. Statistical Analysis

Statistical analysis was performed with the use of MedCalc and Statistica v.12.0 programs. For general characteristics of the studied group of patients after myocardial infarction, mean, minimum and maximum values, standard deviations and percentages were calculated.

In order to assess changes in the examined parameters, in the first stage, the distribution of variables was checked using the Shapiro–Wilk test. In the case of the normal distribution, a parametric test was used to assess the significance of changes in a given group—Student’s *t*-test for dependent samples. However, if the distribution of the results was different from normal, the statistical analysis was performed using the Rank sum tests (Mann–Whitney U test and Wilcoxon test). The level of statistical significance was *p* ≤ 0.05.

## 3. Results

The characteristics of the studied group of patients after myocardial infarction before (A) and after early post-hospital cardiac rehabilitation (A1), including physical activity assessed by the International Physical Activity Questionnaire, are presented in Table 3.

Characteristics of the studied patients after myocardial infarction who did not participate in early post-hospital cardiac rehabilitation (B) and after 3 weeks without rehabilitation (B1) taking into account physical activity assessed by the International Physical Activity Questionnaire is presented in Table 4.

**Figure 1:**
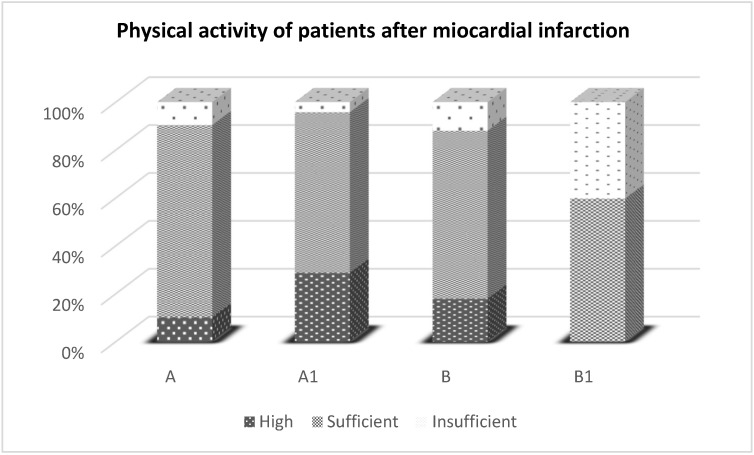
The level of physical activity of the examined patients after myocardial infarction assessed by the International Physical Activity Questionnaire. Explanation abbreviations: A—Studied group of patients after myocardial infarction before rehabilitation, A1—Studied group of patients after myocardial infarction and rehabilitation, B—Studied group patients after myocardial infarction who did not participating in rehabilitation, B1—Examined a group of patients after myocardial infarction and 3 weeks without rehabilitation.

The level of physical activity of the studied patients after myocardial infarction assessed by the International Physical Activity Questionnaire is presented in Figure 1.

In the group of patients after myocardial infarction qualified for early post-hospital cardiac rehabilitation (A) and after its completion (A1), a decrease in the number of people with insufficient physical activity (from 10.2% to 4.2%) was observed, with a simultaneous increase in the number of patients with high levels of physical activity (from 9.6% to 28.7%). On the other hand, in the group of patients after myocardial infarction (B) and after 3 weeks without rehabilitation (B1), none of the respondents achieved a high level of physical activity, whereas the number of people with insufficient physical activity increased (from 11.9% to 40.3%). 

The transcriptional activity of the studied genes: endothelin-1 (ET-1) and endothelin-1 receptor type A (ENDRA) among patients after myocardial infarction before (A) and after early post-hospital cardiac rehabilitation (A1) are presented in Table 5.

Transcriptional activity of the endothelin-1 gene in the studied group of patients after myocardial infarction (A) and after early post-hospital cardiac rehabilitation (A1) increased significantly (*p* = 0.0064).

Comparing the results of the studied patients after myocardial infarction before (A) and after early post-hospital cardiac rehabilitation (A1), no statistically significant differences were observed in the transcriptional activity of the endothelin-1 receptor type A gene (*p* = 0.1410).

Transcriptional activity of the endothelin-1 (ET-1) gene and endothelin-1 receptor type A (ENDRA) in the studied group of patients after myocardial infarction who did not participate in early post-hospital cardiac rehabilitation (B) and after 3 weeks without rehabilitation (B’) are presented in Table 6.

Comparing the results of patients after acute myocardial infarction who did not participate in rehabilitation (B) and after 3 weeks without rehabilitation (B1), a statistically significant increase in the expression of the endothelin-1 gene was observed (*p* = 0.0268).

In the studied group of patients after acute myocardial infarction who did not participate in early post-hospital cardiac rehabilitation (B) and after 3 weeks without rehabilitation (B1), no statistically significant differences in the transcriptional activity of the endothelin-1 receptor type A were observed (*p* = 0.1439). 

The transcriptional activity of endothelin-1 genes and endothelin-1 receptor type A receptor among patients after acute myocardial infarction, taking into account the type of infarction, is presented in Table 7. 

In the studied group of patients after myocardial infarction with ST-segment elevation before and after early post-hospital cardiac rehabilitation, a statistically significant increase in the expression of the endothelin-1 gene was observed (*p* = 0.0234). Moreover, comparing patients with STEMI infarction after rehabilitation to patients after MI with NSTEMI and 3 weeks without rehabilitation, despite the lack of statistically significant difference, the expression of the ET-1 gene was higher among patients after STEMI MI.

It was also observed that the transcriptional activity of the ET-1 gene among patients after acute STEMI infarction after 3 weeks without rehabilitation was significantly higher compared to patients with STEMI after early rehabilitation (*p* = 0.0091). 

The transcriptional activity of the studied genes among patients after myocardial infarction before (A) and after early post-hospital cardiac rehabilitation (A1), including the presence of risk factors, is presented in Table 8, Table 9 and Table 10.

In the studied group of men after myocardial infarction (A) and early post-hospital cardiac rehabilitation (A1), a significant increase in ET-1 expression was observed (*p* = 0.0310).

A statistically significant correlation was observed between the transcriptional activity of the endothelin-1 receptor gene among patients after myocardial infarction before (A) and after early post-hospital cardiac rehabilitation (A1), and the age of the respondents. The correlation was positive (*p* = 0.0001), which means that that expression of the ENDRA genes increased with age. 

Statistically significantly higher transcriptional activity of the ET-1 gene was observed among patients after myocardial infarction and early post-hospital cardiac rehabilitation without diabetes (*p* = 0.0458), but with impaired lipid metabolism (*p* = 0.0107). 

Statistically significantly higher expression of the ET-1 gene was also found among patients after myocardial infarction and post-hospital rehabilitation, compared to non-smokers (*p* = 0.0403).

On the other hand, the transcriptional activity of the endothelin-1 receptor type A was statistically significantly higher both before rehabilitation (*p* = 0.0005) and after rehabilitation (*p* = 0.0001) in the group of patients with acute myocardial infarction and normal body weight, compared to obesity.

## 4. Discussion

In their publication, De Bias et al. paid particular attention to the beneficial effect of physical activity on the integrity of the circulatory system and improvement of the vascular endothelium function among patients with various stages of coronary artery disease [26].

In the studied group of patients after myocardial infarction and early post-hospital cardiac rehabilitation, an increase in physical activity assessed by the International Physical Activity Questionnaire was demonstrated. A significant increase in the physical activity of the studied patients was observed in the work coefficients of a moderate and light nature, with a simultaneous significant reduction in the time spent in a sitting position during the day. On the other hand, the respondents after myocardial infarction, who did not participate in cardiac rehabilitation, spent significantly more time sitting during the day, while spending less time on walking activities. Moreover, none of the examined persons exercised after a heart attack and after 3 weeks without rehabilitation of a severe nature, requiring increased heart rate and breathing. This could be related to the phenomenon of kinesiophobia, which has been described in recent years (the fear of movement of people with cardiovascular diseases) [27].

Physical activity reduction among patients after myocardial infarction increases the risk of cardiovascular disease progression and, consequently, morbidity and mortality. Early post-hospital cardiac rehabilitation enables patients after a heart attack to carry out a controlled effort under the supervision of qualified medical personnel, to learn about the possibilities and limitations of their body in the conditions of the existing disease, and to recognize the disturbing symptoms associated with it [28]. Conducted physical training and education of patients of the discussed group is the initial stage of the treatment process, which should be carried out until the end of life. 

In the presented studies, the transcriptional genes activity of selected vascular endothelial factors among patients after myocardial infarction before and after early post-hospital cardiac rehabilitation and in the group of post-infarction patients not participating in rehabilitation was assessed.

For the study of transcriptional activity, the genes of endothelin-1 (ET-1) and its A-type receptor (ENDRA) acting pro-inflammatory, pro-coagulatory, pro-thrombotic, atherosclerotic and as endothelial vasoconstrictors were selected. 

Other researchers, based on the results of the conducted studies, suggested an increased expression of the endothelin-1 gene, both among patients with coronary artery disease, hypertension, and acute coronary syndrome, compared to healthy subjects [29,30].

In the conducted study, both in the group of patients after myocardial infarction and early post-hospital cardiac rehabilitation, as well as in the group of people not participating in rehabilitation, significantly increased expression of the endothelin-1 gene was demonstrated. It is worth noticing that despite the increased expression of this gene in the studied group of patients after myocardial infarction and early post-hospital cardiac rehabilitation, its transcriptional activity among patients without rehabilitation was even greater. Therefore, it can be concluded that early post-hospital cardiac rehabilitation among patients after myocardial infarction has a positive effect on the transcriptional activity of the ET-1 gene, the reduction in which contributes to the improvement of the vascular endothelial function. 

In addition, it is worth noticing that in the group of patients after myocardial infarction and after early post-hospital cardiac rehabilitation, despite the lack of statistical significance, a decrease in the expression of the endothelin-1 receptor type A (ENDRA) gene was shown, while in the group without rehabilitation it showed an increase. This may be important in explaining the positive effect of physical activity in the studied patients after a heart attack.

Increased transcriptional activity of the endothelin-1 receptor type A gene enhances the unfavorable effect of the cytokine in question on endothelial function. It contributes not only to the increased tension of the blood vessel walls but also, according to some authors, it causes ENDRA mediated growth and proliferation of smooth muscle cells and the triggering of oxidative stress in endothelial cells [31]. In the conducted studies among patients with coronary artery disease, it was shown that blocking the A-type receptor for endothelin-1 improved the function of the vascular endothelium by inhibiting the vasoconstrictor effect of ET-1 [32]. 

It should be emphasized that patients after myocardial infarction, despite the applied revascularization and pharmacological treatment, are not fully cured patients. They often progress to coronary artery disease, develop complications related to it, or recurrent cardiovascular events. It is worth mentioning that the duration of early post-hospital cardiac rehabilitation was on average 3 weeks, so it was relatively short. Therefore, it is extremely important to conduct a multi-stage, comprehensive cardiac rehabilitation which lasts to the end of the patient’s life, provides better treatment results, and contributes to reducing the risk of cardiovascular events and mortality among patients with coronary heart disease and after myocardial infarction.

Confirmation of the sense of the need to conduct the above-mentioned rehabilitation and treatment measures is indicated by, for example, the comparative results of endothelin-1 gene expression among patients after STEMI and early post-hospital rehabilitation with the results of similar patients not participating in rehabilitation, with significantly higher transcriptional activity of the gene mentioned. It is also worth noting that the type of myocardial infarction (STEMI vs. NSTEMI) may play a role in the transcriptional activity of the ET-1 gene under study. Comparing patients after STEMI and rehabilitation with patients with NSTEMI after 3 weeks without rehabilitation, despite the lack of a statistically significant difference, the transcriptional activity of the ET-1 gene was higher among patients with STEMI. Therefore, it can be assumed that the type of myocardial infarction may be a significant factor affecting the success of the rehabilitation process, but it may also result from the pathophysiological basis of myocardial infarction with and without ST-segment elevation.

In their research, Yang et al. have shown that regular physical activity of healthy elderly people, despite the ongoing aging processes, has a beneficial effect on blood vessels and has a protective effect. It should therefore be emphasized that regular physical activity of elderly people, not only healthy people, but most of all those burdened with cardiovascular diseases, is very important [33]. 

On the other hand, A. Chalghouma et al. analyzed the activity of endothelin-1 depending on the presence of risk factors among patients with the diagnosed acute coronary syndrome. They observed that the concentration of ET-1 was significantly higher in the group of men, patients with arterial hypertension, smokers, with normal body weight, and without lipid disorders. Moreover, they found that the level of ET-1 in the patients of the above-mentioned group did not differ between those leading a sedentary lifestyle and those with active and diabetic and non-diabetic patients [30]. In our study, the expression of ET-1 was significantly higher in the group of patients with acute myocardial infarction and one of the following characteristics: without diabetes, with lipid disorders, smoking, and ENDRA with normal body weight. 

The dysfunction of the vascular endothelium precedes the development of coronary disease and may be a prognostic factor for future heart attacks. Understanding the mechanisms of the influence of risk factors on endothelial dysfunction emphasizes their important role in the pathophysiology of many cardiovascular diseases developing on the bases of atherosclerosis. Moreover, each of the risk factors may have a variety of effects on the severity of endothelial dysfunction.

Age over 65 is considered a high-risk factor for cardiovascular diseases. However, it should be added that the increased risk of cardiovascular diseases is associated it is also dependent on gender with age. Literature data indicate that the risk increases in women over 50 years of age and in men over 40 years of age. It is also noted that the definition of “risk age” is not always consistent with the actual age. When determining the “age of risk”, it should be associated with the presence of other risk factors. In the group of men after myocardial infarction and early post-hospital cardiac rehabilitation, a significantly higher transcriptional activity of the endothelin-1 gene was found. This may be due to the unequal size of the groups taking into account the sex of the respondents (19.2% of women and 80.8% of men), which is epidemiologically justified. However, according to gender-specific statistical data, MI is diagnosed more often among men [34]. In the group of men, a greater percentage of patients over 65 was observed compared to the studied women (36% and 28%, respectively). Thus, the increased activity of the ET-1 gene among men could be related to their age. Moreover, a statistically significant correlation was observed between the transcriptional activity of the endothelin-1 receptor gene among patients after myocardial infarction, before and after early post-hospital cardiac rehabilitation, and the age of the respondents. The correlation was positive (*p* < 0.0001), which means that the expression of the ENDRA gene increased with age.

It is known that increased body weight (overweight and obesity) is a risk factor for cardiovascular diseases, including hypertension, lipid metabolism disorders, or diabetes, and has been recognized by the WHO as a civilization disease [35,36]. One of the mechanisms linking it with the diseases mentioned above is increased oxidative stress adversely affecting the function of the endothelium. J.B. Lanier et al. Emphasized that the use of a diet and appropriately adapted physical activity can prevent 1/3 of deaths caused by cardiovascular diseases [37].

In the studied group of patients after MI with normal body weight, both before and after rehabilitation, significantly higher expression of the ENDRA gene was observed compared to obese patients. An interesting observation concerning the above results is the so-called “obesity paradox” described in the literature. Researchers have shown a better prognosis among patients with coronary artery disease and obesity compared to patients with normal body weight [38,39]. It was found that with increasing body mass index (BMI), the percentage of patients and their mortality decreased. The results of the EPIC study also seem to be worth paying attention to, in which it was noted that the lack of physical activity had a much greater effect on increasing the mortality of patients than increased BMI. Thus, in the above-mentioned obesity paradox, the level of cardiovascular and respiratory efficiency may significantly affect the relationship between obesity and the clinical prognosis of patients [40]. Research conducted by M. Matsuda et al. revealed that obese people exhibit systemically higher levels of oxidative stress. The increased amount of adipose tissue and lipid accumulation is a source of reactive oxygen species, contributing to the development of obesity-related diseases, including hypertension, diabetes, and coronary artery disease [41]. 

The negative effect of nicotine on the increase in ET-1 gene expression in various disease entities has been confirmed by scientific studies [41,42]. As a result of the action of nicotine in the body, there is an increased production of reactive oxygen species, intensification of oxidative processes, lipid oxidation, and a decrease in the bioavailability of nitric oxide and impaired vasodilator functions. In terms of cardiovascular disease prevention, it has been shown that most of the above-mentioned processes are completely reversible as a result of smoking cessation. In our research, statistically, significantly higher expression of the ET-1 gene was found among patients after myocardial infarction and post-hospital rehabilitation, compared to non-smokers (*p* = 0.0403). Moreover, it was found that early post-hospital cardiac rehabilitation did not change the transcriptional activity of genes of the studied vascular endothelial factors in diabetic patients. A study by L. J. Reynolds et al. showed a correlation between elevated blood glucose concentration and increased endothelin-1 activity. Thus, the lack of changes in the activity of the ET-1 gene among patients with diabetes could have resulted from the use of prescribed medications and/or diet [43].

## 5. Conclusions

A smaller increase in the expression of the endothelin-1 gene with a simultaneous increase in physical activity in the group of patients after early post-hospital cardiac rehabilitation, compared to patients not participating in it, proves the beneficial effect of rehabilitation and indicates the need for its use throughout life. 

The increase in the transcriptional activity of the endothelin-1 gene in the group of men, smokers, and people with lipid metabolism disorders, and among patients after myocardial infarction with ST-segment elevation (STEMI), as well as endothelin-1 receptor type A (ENDRA) with the age of the respondents may indicate another pathomechanism of pro-atherogenic activity of the above-mentioned factors.

## Figures and Tables

**Table 1:** Characteristic of the studied group.

Variable		Studied Group						
		AB *n* = 234; 100%		A *n* = 167; 71.4%		B *n* = 67; 28.6%		*p*
Sex	Women	*n* = 45; 19.2%		*n* = 35; 21%		*n* = 10; 15%		
	Men	*n* = 189; 80.8%		*n* = 132; 79%		*n* = 57; 85%		
Mean x, Standard Deviation ± SD		x	±SD	x	±SD	x	±SD	
Age [y]		60.29	9.39±	60.13	9.78±	60.68	8.41±	0.687
Weight [kg]		81.04	11.57±	80.57	12.22±	82.21	9.76±	0.329
Hight [m]		1.72	0.07±	1.72	0.07±	1.73	0.05±	0.546
BMI [kg/m^2^]		27.18	3.27±	27.09	3.29±	27.41	3.23±	0.513

Explanation of abbreviations: *n*—Number, x—mean, SD—Standard deviation, *p*—Statistically significant difference, A—Studied group of patients after myocardial infarction and rehabilitation, B—Studied group of patients after myocardial infarction without rehabilitation, BMI—Body mass index.

**Table 2:** The characteristics of the studied group of patients after myocardial infarction participating in early post-hospital cardiac rehabilitation (A), including the rehabilitation model.

Studied Group of Patients A, *n* = 167, 100%			
Variable		*n*	%
Model of cardiac rehabilitation	A	93	55.69%
	B	66	39.52%
	C	8	4.79%
	D	0	0%

Explanation of abbreviations: *n*—Number, %—Percentage of group, A—Studied group of patients after heart attack before rehabilitation.

**Table 3:** Characteristics of the studied group of patients after myocardial infarction before (A) and after early post-hospital cardiac rehabilitation (A1), including physical activity assessed by the International Physical Activity Questionnaire.

Studied Group of Patients A, A1 *n* = 167; 100%					
Variable	Group				*p*
	A		A1		
**AF1 [MET]**	x = 21.55	±SD = 72.51	x = 41.67	±SD = 191.29	0.07
**AF2 [MET]**	x = 359.28	±SD = 461.52	x = 385.51	±SD = 36.99	0.001
**AF3 [MET]**	x = 1607.71	±SD = 705.55	x = 1631.43	±SD = 705.27	0.010
**Time spent sitting [min.]**	x = 236.73	±SD = 130.35	x = 228.31	±SD = 115.36	0.012

Explanation of abbreviations: *n*—Number, A—Studied group of patients after myocardial infarction before rehabilitation, A1—Studied group of patients after myocardial infarction and rehabilitation, x—Average, MET—Metabolic equivalent, AF1—Heavy effort, AF2—Moderate effort, AF3—Light effort, min.—Minutes, *p*—Statistically significant difference.

**Table 4:** Characteristics of the studied group of patients after myocardial infarction who did not participate in early post-hospital cardiac rehabilitation (B) and after 3 weeks without rehabilitation (B1), taking into account physical activity assessed by the International Physical Activity Questionnaire.

Studied Group of Patients B, B1 *n* = 67; 100%					
Variable	Group				*p*
	B		B1		
**AF1 [MET]**	x = 124	±SD = 301.92	x = 102.86	±SD = 250.29	0.103
**AF2 [MET]**	x = 616.19	±SD = 615.03	x = 552.38	±SD = 475.64	0.109
**AF3 [MET]**	x = 1613.86	±SD = 860.79	x = 1426.63	±SD = 496.19	0.009
**Time spent sitting [min.]**	x = 208	±SD = 93.39	x = 218	±SD = 83.36	0.03

Explanation of abbreviations: *n*—Number, B—Studied group of patients after myocardial infarction who did not participate in early post-hospital cardiac rehabilitation, B1—Studied group of patients after myocardial infarction after 3 weeks without rehabilitation, x—Average, MET—Metabolic equivalent, AF1—Heavy effort, AF2—Moderate effort, AF3—Light effort, min.—Minutes, *p*—Statistically significant difference.

**Table 5:** Transcriptional activity of the endothelin-1 gene and endothelin-1 receptor type A among patients after myocardial infarction heart before (A) and after early post-hospital cardiac rehabilitation (A1).

Studied Group of Patients A, A1 *n* = 167; 100%	
**ET-1 A vs. ET-1 A1**	*p* = 0.0064
**ENDRA A vs. ENDRA A1**	*p* = 0.1410

Explanation of abbreviations: ET-1—Endothelin-1, ENDRA—Endothelin-1 receptor type A, A—Transcriptional activity of the studied gene among patients after myocardial infarction before rehabilitation, A1—Transcriptional activity of the studied gene among patients after myocardial infarction and rehabilitation, *p*—statistically significant difference.

**Table 6:** Transcriptional activity of the endothelin-1 gene and its A-type receptor among patients after MI who did not participate in early post-hospital cardiac rehabilitation (B) and after 3 weeks without rehabilitation (B’).

Studied Group of Patients B, B1 *n* = 67; 100%	
**ET-1 B vs. ET-1 B1**	*p* = 0.0268
**ENDRA B vs. ENDRA B1**	*p* = 0.1439

Explanation of abbreviations: ET-1—Endothelin-1, ENDRA—Endothelin-1 receptor type A, B—Studied group of patients after myocardial infarction who did not participate in early post-hospital cardiac rehabilitation, B1—Studied group of patients after myocardial infarction and after 3 weeks without rehabilitation, *p*—Statistically significant difference.

**Table 7:** Transcriptional activity of endothelin-1 genes and endothelin-1 receptor type A receptor in the studied group of patients after myocardial infarction, taking into account the type of myocardial infarction.

Type of Miocardial Infraction	Genes	
	ET-1	ENDRA
**STEMI** A vs. **STEMI** A**1**	*p* = 0.0234	*p* = 0.4623
**NSTEMI** A vs. **NSTEMI** A**1**	*p* = 0.2832	*p* = 0.0799
**STEMI** A vs. **NSTEMI** A	*p* = 0.7774	*p* = 0.2937
**STEMI** A**1** vs. **NSTEMI** A**1**	*p* = 0.2456	*p* = 0.2302
**STEMI** A**1** vs. **NSTEMI** B**1**	*p* = 0.9176	*p* = 0.7452
**STEMI** A**1** vs. **STEMI** B**1**	*p* = 0.0091	*p* = 0.1100
**NSTEMI** A**1** vs. **NSTEMI** B**1**	*p* = 0.4653	*p* = 0.2000

Explanation of abbreviations: ET-1—Endothelin-1, ENDRA—Endothelin-1 receptor type A, STEMI—Myocardial infarction with ST segment elevation, NSTEMI—Myocardial infarction without ST segment elevation, A—Studied group of patients after myocardial infarction before rehabilitation, A1—Studied group of patients after myocardial infarction and rehabilitation, B1—Studied group of patients after myocardial infarction and after 3 weeks without rehabilitation, *p*—Statistically significant difference.

**Table 8:** Transcriptional activity of endothelin-1 genes and type 1 endothelin receptor in the studied group of patients after myocardial infarction before (A) and after early post-hospital cardiac rehabilitation (A1) taking into account the sex of the respondents.

Variable	Genes	
	ET-1	ENDRA
**W** (A) vs. **W** (A**1**)	*p* = 0.2436	*p* = 0.7931
**M** (A) vs. **M** (A**1**)	*p* = 0.0310	*p* = 0.5321
**W** (A) vs. **M** (A**1**)	*p* = 0.4743	*p* = 0.5619
**W** (A1) vs. **M** (A**1**)	*p* = 0.4554	*p* = 0.1005

Explanation of abbreviations: W—Women, M—Men, A—Studied group of patients after myocardial infarction before rehabilitation, A1—Studied group of patients after myocardial infarction and rehabilitation, ET-1—Endothelin-1, ENDRA—Type 1 endothelin receptor A, *p*—Statistically significant difference.

**Table 9:** Transcriptional activity of endothelin-1 genes and its A-type receptor among patients after myocardial infarction before (A) and after early post-hospital cardiac rehabilitation (A1) taking into account age.

	Genes	
Under Study Parameter	Variable ET-1	Variable ENDRA
**Patients age** **A vs. A1**	R = 0.225 *p* = 0.1135	R = 954 *p* < 0.0001

Explanation of abbreviations: ET-1—Endothelin-1, ENDRA—Endothelin-1 receptor type A, A—Transcriptional activity of the studied gene among patients after myocardial infarction before rehabilitation, A1—Transcriptional activity of the studied gene among patients after myocardial infarction and rehabilitation, R—Correlation coefficient, *p*—Statistically significant difference.

**Table 10:** Transcriptional activity of the studied genes among patients after myocardial infarction before (A) and after early post-hospital cardiac rehabilitation (A1) taking into account the factors risk.

Risk Factors		Genes	
		ET-1	ENDRA
**Arterial hypertension**	**HA (−)** A vs. **HA (−)** A**1**	*p* = 0.0735	*p* = 0.2446
	**HA (+)** A vs. **HA (+)** A**1**	*p* = 0.1227	*p* = 0.2195
	**HA (−)** A vs. **HA (+)** A	*p* = 0.1329	*p* = 0.4524
	**HA (−)** A**1** vs. **HA (+)** A**1**	*p* = 0.5237	*p* = 0.7327
**Diabetes**	**DM (−)** A vs. **DM (−)** A**1**	*p* = 0.0458	*p* = 0.1492
	**DM (+)** A vs. **DM (+)** A**1**	*p* = 0.1648	*p* = 0.3901
	**DM (−)** A vs. **DM (+)** A	*p* = 0.3085	*p* = 0.2807
	**DM (−)** A**1** vs. **DM (+)** A**1**	*p* = 0.0814	*p* = 0.2189
**Obesity**	**NBW** A vs. **NBW** A**1**	*p* = 0.0734	*p* = 0.0712
	**O** A vs. **O** A**1**	*p* = 0.0906	*p* = 0.3104
	**NBW** A vs. **O** A	*p* = 0.3732	*p* = 0.0005
	**NBW** A1 vs. **O** A**1**	*p* = 0.9005	*p* = 0.0001
**Smoking**	**S(−)** A vs. **S (−)** A**1**	*p* = 0.0573	*p* = 0.1410
	**S (+)** A vs. **S (+)** A**1**	*p* = 0.0636	*p* = 0.6411
	**S (−)** A vs. **S (+)** A	*p* = 0.1584	*p* = 0.0523
	**S (−)** A**1** vs. **S (+)** A**1**	*p* = 0.0403	*p* = 0.1196
**Lipid disorders**	**LD (−)** A vs. **LD** **(−)** A**1**	*p* = 0.0495	*p* = 0.4477
	**LD (+)** A vs. **LD (+)** A**1**	*p* = 0.0107	*p* = 0.1788
	**LD (−)** A vs. **LD (+)** A	*p* = 0.9799	*p* = 0.6122
	**LD (−)** A1 vs. **LD (+)** A**1**	*p* = 0.2950	*p* = 0.3353

Explanation of abbreviations: ET-1—Endothelin-1, ENDRA—Endothelin-1 receptor type A, A—Studied group of patients after myocardial infarction before rehabilitation, A1—Studied group of patients after myocardial infarction and rehabilitation, HA—Arterial hypertension, DM—Diabetes, O—Obesity, NBW—Normal body weight, S—Smoking, LD—Lipid disorders, (+)—Factor present, (−)—Factor absent, *p*—Statistically significant difference.
